# Regulatory T Cell Suppression of Gag-Specific CD8^+^ T Cell Polyfunctional Response After Therapeutic Vaccination of HIV-1-Infected Patients on ART

**DOI:** 10.1371/journal.pone.0009852

**Published:** 2010-03-24

**Authors:** Bernard J. C. Macatangay, Marta E. Szajnik, Theresa L. Whiteside, Sharon A. Riddler, Charles R. Rinaldo

**Affiliations:** 1 Division of Infectious Diseases, University of Pittsburgh School of Medicine, Pittsburgh, Pennsylvania, United States of America; 2 Department of Pathology, University of Pittsburgh Cancer Institute, Pittsburgh, Pennsylvania, United States of America; 3 Department of Infectious Diseases and Microbiology and Department of Pathology, University of Pittsburgh Graduate School of Public Health and School of Medicine, Pittsburgh, Pennsylvania, United States of America; University of Sao Paulo, Brazil

## Abstract

We tested the hypothesis that therapeutic vaccination against HIV-1 can increase the frequency and suppressive function of regulatory, CD4^+^ T cells (Treg), thereby masking enhancement of HIV-1-specific CD8^+^ T cell response. HIV-1-infected subjects on antiretroviral therapy (N = 17) enrolled in a phase I therapeutic vaccine trial received 2 doses of autologous dendritic cells (DC) loaded with HIV-1 peptides. The frequency of CD4^+^CD25^hi^FOXP3^+^ Treg in blood was determined prior to and after vaccination in subjects and normal controls. Polyfunctional CD8^+^ T cell responses were determined pre- and post-vaccine (N = 7) for 5 immune mediators after in vitro stimulation with Gag peptide, staphylococcal enterotoxin B (SEB), or medium alone. Total vaccine response (post-vaccine–pre-vaccine) was compared in the Treg(+) and Treg-depleted (Treg-) sets. After vaccination, 12/17 subjects showed a trend of increased Treg frequency (P = 0.06) from 0.74% to 1.2%. The increased frequency did not correlate with CD8^+^ T cell vaccine response by enzyme linked immunosorbent assay for interferon γ production. Although there was no significant change in CD8^+^ T cell polyfunctional response after vaccination, Treg depletion increased the polyfunctionality of the total vaccine response (P = 0.029), with a >2-fold increase in the percentage of CD8^+^ T cells producing multiple immune mediators. In contrast, depletion of Treg did not enhance polyfunctional T cell response to SEB, implying specificity of suppression to HIV-1 Gag. Therapeutic immunization with a DC-based vaccine against HIV-1 caused a modest increase in Treg frequency and a significant increase in HIV-1-specific, Treg suppressive function. The Treg suppressive effect masked an increase in the vaccine-induced anti-HIV-1-specific polyfunctional response. The role of Treg should be considered in immunotherapeutic trials of HIV-1 infection.

## Introduction

The ability of regulatory T cells (Treg) to control the immune response to infections can have serious implications in the body's capability to eradicate invading pathogens. The normal physiologic function of Treg is to mediate peripheral tolerance, thereby preventing autoimmunity [1], and to limit the damage caused by inflammatory responses to infectious agents. However, Treg-mediated suppression can also lead to the inhibition of antimicrobial responses and in so doing, allow persistent infections.

Although the frequency and function of Treg in the peripheral blood and lymphatic tissues of patients with HIV-1 infection have been explored, no consistent results have emerged. It is also uncertain whether Treg mainly protect against or contribute to persistent HIV-1 infection and consequent disease progression. A number of studies have shown an increase in the frequency of Treg in HIV-1-infected individuals particularly in those with untreated infection and in those with detectable plasma viremia. [2]–[4] Such increases in Treg could potentially interfere with functions of antiviral CD8^+^ T lymphocytes and thus contribute to persistent HIV-1 infection. Furthermore, as the CD4^+^ T cell population decreases in advanced disease, the absolute number of Treg has also been shown to proportionately decline. [2] Others have followed Treg changes over a period of 6 months, and noted a significant Treg decrease in the untreated HIV-1-infected population compared to those who were virally suppressed. [5] This depletion of Treg was associated with immune activation [6], which has been shown to be a predictor of disease progression. [7] In this context, depletion in the number or function of Treg appears to contribute to HIV-1 progression. However, a nested case-control study by Cao et al. [8] showed significant expansion of Treg and increase in T cell activation in subjects with rapidly progressing HIV-1 infection. These conflicting results might reflect methodologic differences in identifying human Treg, as no universally acceptable marker has been defined for these cells. Since the FOXP3 transcription factor is not universally correlated with regulatory activity in humans [1], it cannot be reliably used for human Treg identification. Additionally, cross-sectional studies have a limited value in comparison with longitudinal analyses of Treg. Despite the inconsistent frequency results, functional studies have demonstrated that Treg isolated from both peripheral blood and lymphoid tissues of HIV-1 infected individuals show strong suppressor activity and that they suppress HIV-1-specific effector functions of T cells. [2], [9], [10] Specifically, Treg isolated from HIV-infected patients have been shown to suppress HIV-1-specific CD8^+^ T cell interferon γ (IFNγ) and tumor necrosis factor α (TNFα) secretion as well as Gag-specific cytolytic activity. [11]

We have completed a phase I therapeutic vaccination trial in which autologous dendritic cells (DC) loaded with HIV-1 peptides representing immunodominant epitopes were delivered either subcutaneously or intravascularly to subjects with chronic HIV-1 infection on antiretroviral therapy (ART). [12] The ultimate goal of this approach is to elicit robust immune responses against the virus, resulting in a control of viral replication and prevention of disease progression. We demonstrated feasibility and safety of this vaccine and observed a modest and transient increase in the number of T cells producing HIV-1-peptide-specific IFNγ as detected by the enzyme linked immunosorbent (ELISPOT) assay. We hypothesized that the observed responses were modest because the vaccine for HIV-1 designed to increase the immune response to the virus, had led to an increase in the frequency and suppressive function of Treg resulting in down modulation of the response. To test this hypothesis, we evaluated Treg frequency and their ability to suppress HIV-1 specific polyfunctional CD8^+^ T cell response using cryopreserved blood specimens from the vaccinated patients. This is because studies have shown that an effective immune response against HIV is characterized by the ability of CD8^+^ T cells to produce multiple immune mediators: IFNγ, interleukin-2 (IL2), TNFα, macrophage inflammatory protein 1β(MIP-1β), and the cytotoxic degranulation molecule CD107a. [13] These responses have been demonstrated in HIV-1-infected individuals who are able to control the virus without ART and directly correlate with CD4 counts and inversely correlate with viremia and disease progression. [14]–[16]

## Materials and Methods

### Ethics Statement

Peripheral blood mononuclear cells (PBMC) used in this study were obtained from the participants of the therapeutic DC vaccine study reported by Connolly et al. [12] This study was approved by the University of Pittsburgh Institutional Review Board. A written informed consent was signed by all the subjects including the consent to use the cryopreserved PBMC for further immunologic studies.

### Study Population

Eighteen HIV-1-infected, HLA A*0201 positive subjects with suppression of plasma HIV-1 RNA (<50 copies/mL) on a stable antiretroviral regimen were included in the study. Briefly, the subjects received 2 doses (either subcutaneous or intravenous) of a vaccine composed of autologous DC. The monocyte-derived DCs were generated by culturing monocytes, obtained by leukapheresis, with IL-4 and granulocyte-monocyte colony stimulating factor (GM-CSF), then matured with IL-6, IL-1β, and TNF-α, and finally loaded with synthetic peptides Gag (362–370, VLAEAMSQV) (VV9), Pol (464–472, ILKEPVHGV) (IV9), Env (121–129, KLTPLCVTL) (KL9), which are three highly conserved, HIV-1, immunodominant, HLA A*0201-restricted peptides, and an influenza A matrix protein peptide (58–66, GILGFVFTL) (GL9). These epitope sequences were based on HIV-1 strain HXB2 in the Los Alamos HIV Molecular Immunology Database (http://www.hiv.lanl.gov/content/immunology/index.html). It should be noted that these amino acid positions differ from those previously published [12] which were based on Altfeld et. al. [17]. The two vaccine doses were given 2 weeks apart. The immune responses to the vaccine were evaluated in an ELISPOT assay for IFNγ production. PBMC obtained from 17 subjects who received both vaccine doses were used. Polyfunctional studies were performed on PBMC from 7 subjects with available samples.

### Evaluation of CD8^+^ T cell Polyfunctionality

Cryopreserved PBMC from 7 subjects were obtained at baseline and at 2 weeks after the second vaccine dose, and were thawed for intracellular cytokine staining (ICS) and analyzed by flow cytometry in order to measure the expression of the following immune mediators: IFNγ, TNFα, IL-2, MIP-1β, and CD107a. [18]–[20] Cells were rested in media overnight at 37°C and then washed. Aliquots of 10^6^ cells from paired pre- and post-vaccine samples were then co-cultured with Gag peptide (VV9, 10 µL at 0.1 mg/mL), anti-CD28/49d monoclonal antibody (mAb) (2 µL at 1 µg/mL, BD Bioscience), anti-CD107α mAb PE-Cy5 (20 µL, BD Pharmingen), monensin (2 µL of 5 µg/mL, Sigma), brefeldin A (2 µL of 5 µg/mL, BD Bioscience) and PBS (14 µL) in a 96-well plate. The negative control was medium only and the positive control was cells stimulated with *Staphylococcus* enterotoxin B (SEB) (4 µL of 50 µg/mL, Sigma). Plates were incubated at 37°C for 6 h and then at 4°C for 16 h. [19] Cells were washed and then simultaneously stained with anti-CD3 mAb PE-Cy7 (5 µL; BD Pharmingen), anti-CD8 mAb APC-Cy7 (5 µL; Biolegend), and anti-CD4 mAb AF 700 (5 µl/; BD Pharmingen), incubated in the dark for 30 minutes at room temperature (RT), and then washed. BD FACS Lysing solution (100 µl/well) was added and incubated for 10 minutes at RT. The plates were washed with cold buffer, treated with BD FACS Permeabilizing Solution 2 (200 µl/well), incubated for 10 minutes at RT, and then washed with 200 µl/well of the cold buffer. The following mAb were then added: anti-TNFα PB (20 µL, eBioscience), anti-MIP-1β PE (5 µL, BD Pharmingen), anti-IL2 APC (5 µL, Biolegend), and anti-IFNγ FITC (1 µL, BD Pharmingen). The plates were incubated in the dark for 30 min, washed with 150 µl/well of cold buffer, and fixed with 1% paraformaldehyde in PBS. Cells were analyzed using a LSR-II 12-color flow cytometer (BD Biosciences). The total vaccine response for each subject was obtained by subtracting pre-vaccine from post-vaccine responses, that is, subtracting the pre-vaccine percentages of CD8^+^ T cells secreting one or a combination of immune mediators from the post-vaccine percentages. The total vaccine response represents the vaccine-induced change in CD8^+^ T cell immune mediator secretion from baseline. Polyfunctional responses of CD8^+^ T cells pre- and post-vaccine and the total vaccine response were assessed using the SPICE program (Version 4.3, Mario Roederer, Vaccine Research Center, NIAID, NIH).

### Immunophenotyping and Treg Frequency

**Figure 1:**
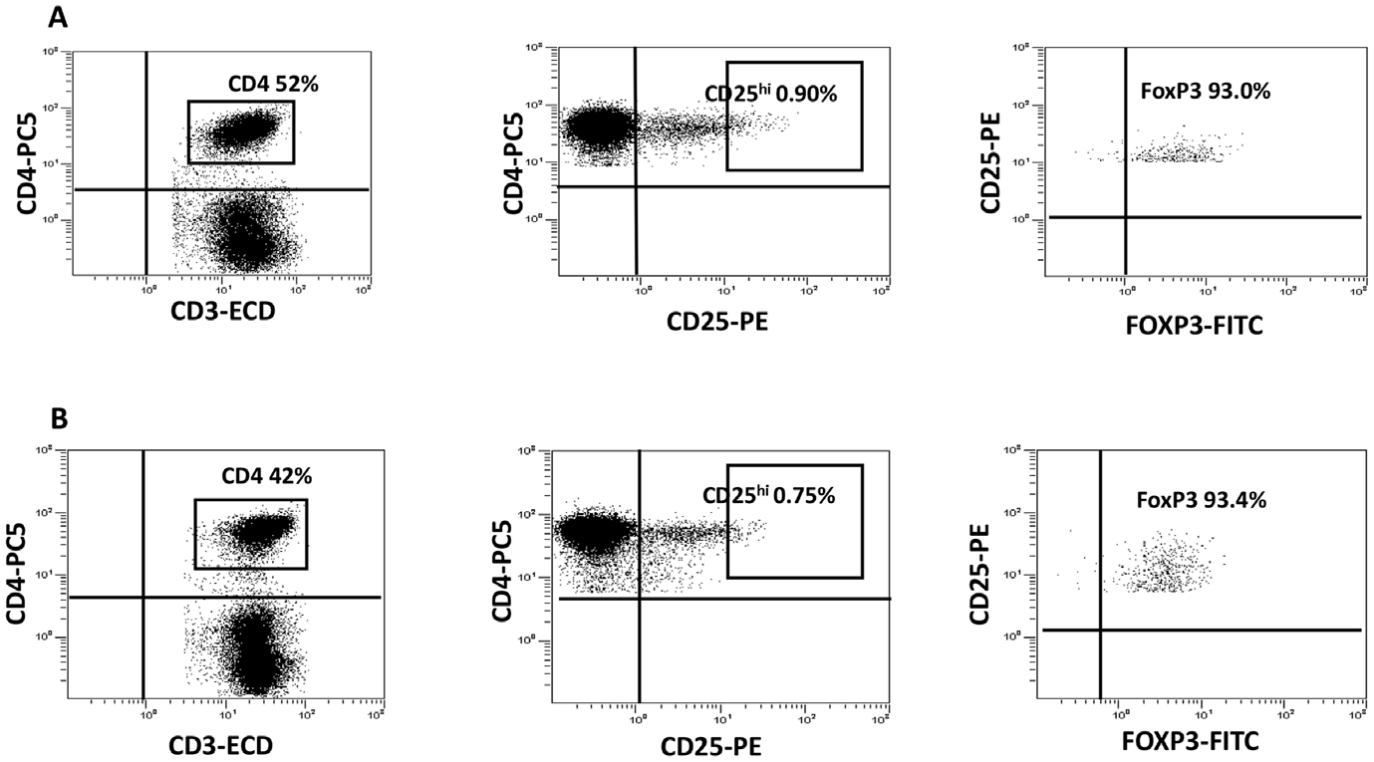
Gating of Treg in representative subjects. Shown above is the gating used to determined the Treg frequency in a representative normal control (A) and an HIV-1-infected subject, pre-vaccine (B). The Treg population was defined as CD3^+^CD4^+^CD25^HI^FOXP3^+^.

We obtained PBMC from stored samples of all 17 subjects at baseline, two weeks post-vaccination (week 6), and 12 weeks post-vaccination (week 16). Cryopreserved cells were thawed, placed in 5 different tubes (per subject), and stained with 5 µl each of anti-CD3 mAb ECD, anti-CD4 mAb PC5, anti-CD25 mAb PE (Beckman Coulter), and the following mAb, one in each tube: anti-CD45RO FITC, anti-glucocorticoid-induced tumor necrosis factor receptor (GITR) FITC (Beckman Coulter). For the other two tubes, cells were first stained with anti-CD3, anti-CD4, and anti-CD25 mAb, and then they were permeabilized with 300 µL of 1% saponin for 30 minutes, washed, stained with 5 µl each of anti-FOXP3 mAb FITC (eBioscience) and anti-cytotoxic T-lymphocyte antigen 4 (CTLA-4) mAb PE (Beckman Coulter), and then incubated in the dark for another 30 minutes. In the tube containing cells stained with anti-CTLA4 PE, anti-CD25 FITC was used. Cells were then washed and fixed with 1% paraformaldehyde in PBS and then analyzed using a 4-color flow cytometer (Beckman Coulter). PBMC obtained from 10 HIV-1 negative controls (NC) were also phenotyped for comparison. The Treg population was identified as CD3^+^CD4^+^CD25^hi^FOXP3^+^ (Fig. 1), and percentages of this population were determined for each time point in every subject. The mean of five replicates of the Treg population was used as the Treg frequency for a particular subject. CD3^+^CD4^+^CD25^hi^ phenotypic profiles were compared for the expression of CD45RO, FOXP3, GITR, and CTLA4. The pre- to post-vaccine changes in the Treg frequency were correlated with the levels of anti-HIV-1 immune response measured in an ELISPOT assay. A positive response in the assay was defined as a response of >10 spots per 10^5^ PBMC, background subtracted, to any of the three HIV-1 peptides, 2 weeks after the second vaccine dose. Pre-vaccine responses greater than post-vaccine responses were considered as no response.

### Evaluation of Treg Suppressive Function

Stored PBMC were obtained pre- and post-vaccine from the 7 subjects on whom polyfunctional studies were done. The population of Treg was depleted using two different methods: in three subjects, we removed the CD4^+^CD25^+^ Treg using a magnetic bead separation system (AutoMACS cell separation, Miltenyi Biotec), as per manufacturer's instructions, while in the other 4 subjects, we used a flow-based cell sorter (Beckman Coulter MoFlo) to separate the CD4^+^CD25^hi^ T cells. The remaining cells were then evaluated for CD8^+^ T cell polyfunctionality using the methods described above. The polyfunctionality of the total vaccine response (post-vaccine–pre-vaccine response) with and without Treg depletion were then compared using SPICE.

### Statistical analysis

Two-tailed signed rank test was used to compare Treg frequencies between the time points. Comparisons between the immunophenotype of NC, pre-, and post-vaccine were made using Kruskal-Wallis and Mann-Whitney *U* tests. Analysis of flow cytometry data and creation of Boolean combinations of single functional gates were done using BD Diva software. All data were background subtracted. Total vaccine response was obtained by subtracting the pre-vaccine response from the post-vaccine response. Statistical analysis of the Boolean-gated data used in the polyfunctional responses was done using the SPICE software that uses a permutation comparison test, based on chi-square statistics, for comparison between pie charts.

## Results

### Demographics and Vaccine Response

The baseline characteristics of the subjects have been previously described. [12] Briefly, 14/17 subjects who were included in this study were male. The median CD4^+^ T cell count nadir was 259 while the median pre-vaccine CD4^+^ T cell count was 717 cells/mm^3^. All of the subjects were on a stable antiretroviral regimen and had an HIV RNA of <50 copies/mL. Based on our definition of a positive immune response in IFNγ production by ELISPOT, 6/17 subjects were considered responders to at least one peptide, two of whom had a positive response to Gag peptide.

**Figure 2:**
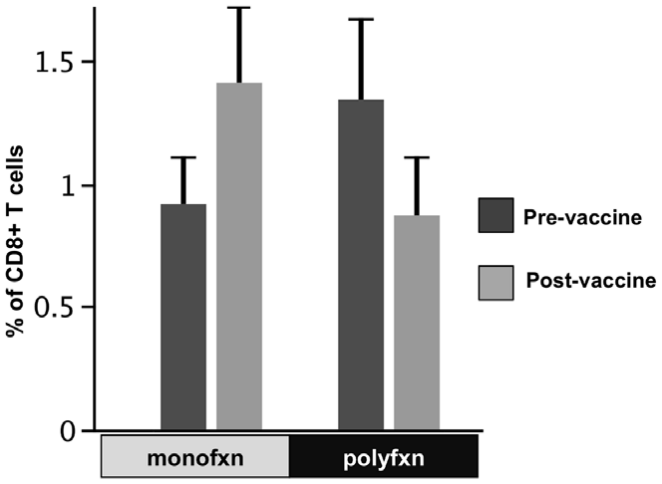
Percentages of CD8^+^ T cells with poly vs monofunctional responses before and after vaccination. Bars are the mean percentages (+SE) of CD8^+^ T cells in paired pre- and post-vaccine samples secreting one (monofxn) or more than one (polyfxn) immune mediators (IFNγ, TNFα, IL-2, MIP-1β, and CD107a) in response to Gag peptide. (P = NS; N = 7)

Polyfunctional response of CD8^+^ T cells to the vaccine using our flow-based assay was evaluated in 7 subjects, 4 of whom were vaccine responders via the ELISPOT assay (data not shown). Polyfunctional responses were detected in all 7 subjects in both pre- and post-vaccine samples using ICS (Fig. 2). There was no significant change in the pattern of polyfunctional response before and after the vaccine. In fact, the pre-vaccine polyfunctional responses appeared to be more extensive than those observed after vaccine administration, implying no vaccine response (Fig. 2). All three non-responders by the ELISPOT assay showed IFNγ production using the ICS, and in 2/3 non-responders, we detected post-vaccine CD8^+^ T cells secreting IFNγ plus one other immune mediator (data not shown).

### Treg Frequencies and Immunophenotype

**Figure 3:**
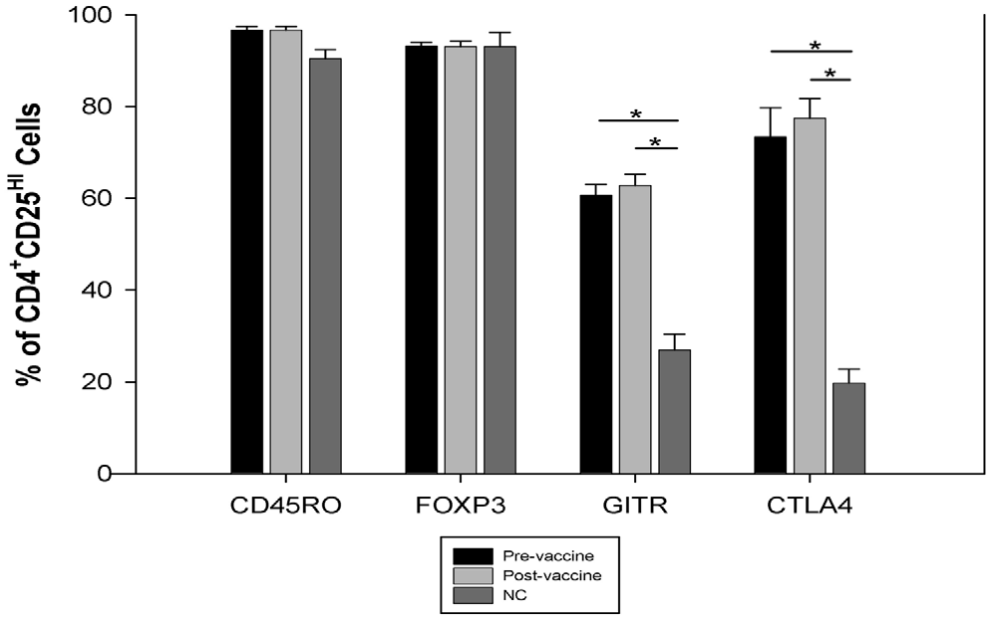
Comparison of CD45RO, FOXP3, GITR, and CTLA4 expression. The mean percentage expression in CD4^+^CD25^HI^ T cells was compared among normal control (NC; N = 10) and the HIV-1-infected subjects pre- and post-vaccine (N = 17). CD4^+^CD25^HI^ expression of GITR and CTLA4 in HIV-1-infected subjects (pre- and post-vaccine) was higher than in NC. (P<0.01)

**Figure 4:**
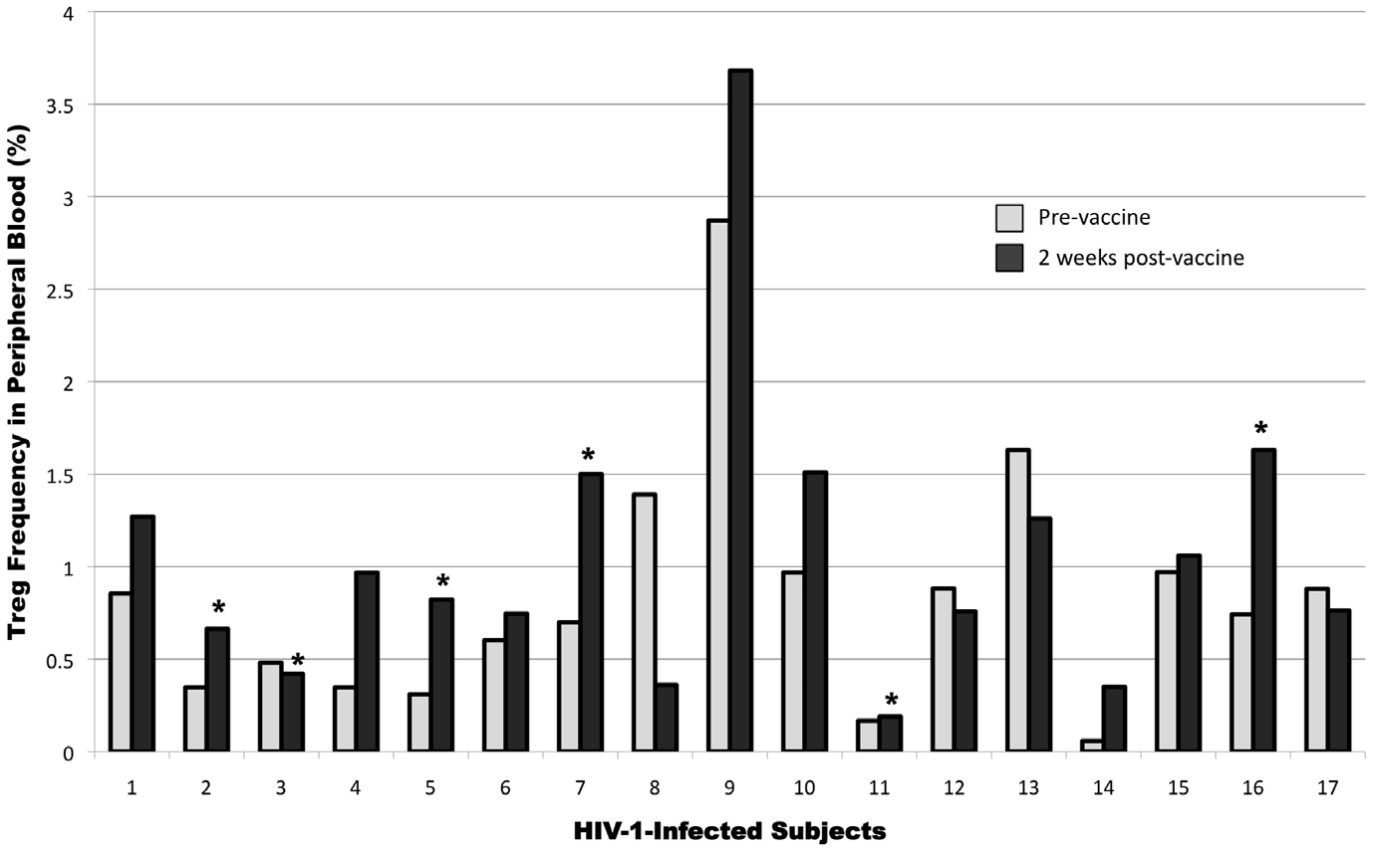
Treg frequency in paired pre- and post-vaccine of the 17 subjects. Percentages of CD4^+^CD25^HI^FOXP3^+^ T cells before (pre-vaccine) and 2 weeks after therapeutic vaccination in each of the 17 HIV-1-infected subjects are shown. Asterisks indicate subjects who had a positive vaccine response by ELISPOT analyses. There was a trend of increased Treg frequency post-vaccine. (P = 0.06)

In order to determine whether the Treg population had any influence on the modest responses to the vaccine, we measured the pre- and post-vaccine Treg frequency using flow cytometry in the 17 subjects. Treg gating of a representative HIV-1-infected subject and NC are shown in Fig. 1. The mean frequency of Treg in the CD3^+^CD4^+^ subset was 0.84% in the subjects pre-vaccine, as compared to 0.78% in NC. Prior to vaccination, the Treg subset in HIV-1-infected subjects was enriched in CTLA4 (20% in NC vs. 73% in subjects; P<0.01) and GITR (27% in NC vs. 61%; P<0.01) relative to NC (Fig. 3). However, pre- and post-vaccine Treg expressions of both CTLA4 and GITR in the HIV-1 subjects were similar. Percent expression of CD4^+^CD25^hi^ cells for FOXP3 and CD45RO was similar among NC and study subjects pre- and post-vaccine. After vaccination, 12/17 (70.6%) subjects showed an increasing trend in the frequencies of Treg (from 0.74% to 1.2%; P = 0.06) with the median increase of 30% (Fig. 4). In 8/12, the Treg frequency increased by more than 50% from the pre-vaccine value. Of the 11 who were considered vaccine non-responders by ELISPOT assay, 7 (64%) had an increase in the Treg frequency. There was no correlation between changes in the pre- to post-vaccine Treg frequency and the ELISPOT responses.

### Treg Suppressive Function

**Figure 5:**
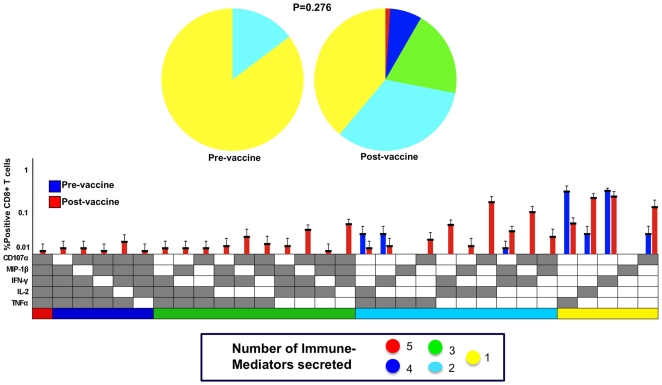
Polyfunctional response of CD8^+^ T cells pre- and post-vaccine after Treg depletion. The response patterns are color-coded by the number of positive functions. The x-axis contains the combinations of positive immune mediators whereas the y-axis is the percentage of CD8^+^ T cells secreting each combination. The pie charts show the proportion of CD8^+^ T cells that are polyfunctional, i.e., secreting more than 1 immune mediator in response to stimulation with Gag peptide. There was increased polyfunctionality after the Treg were removed. Despite the apparent increase in polyfunctionality, the permutation comparison test showed no statistical difference between the two pie charts. (P = 0.276; N = 7)

To evaluate suppressive function, we measured the polyfunctional responses of CD8^+^ T cells to Gag peptide (VV9) before and after depletion of Treg from both pre- and post-vaccine PBMC in 7 subjects (Fig. 5). Upon removal of Treg, CD8^+^ T cell polyfunctionality increased in the post-vaccine samples with a greater number of the CD8^+^ T cells secreting multiple immune mediators. Although there was an apparent increase in the polyfunctional T cell response pattern post-vaccination, the permutation comparison test was unable to detect a significant difference between pre- and post-vaccine responses (P = 0.276).

**Figure 6:**
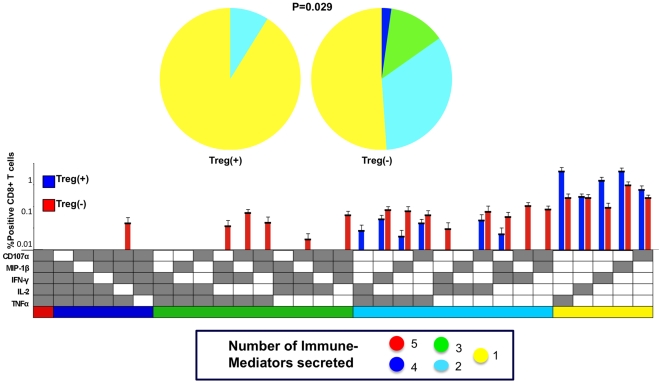
Polyfunctionality of the total vaccine response with (Treg+) and without Treg (Treg−). The total vaccine response was obtained by subtracting the pre-vaccine response from the post-vaccine response and represents the vaccine-induced change in CD8^+^ T cell secretion of immune mediators from baseline. Analysis by permutation comparison showed a significant increase in polyfunctional response to Gag peptide in the samples depleted of Treg. (P = 0.029; N = 7)

**Figure 7:**
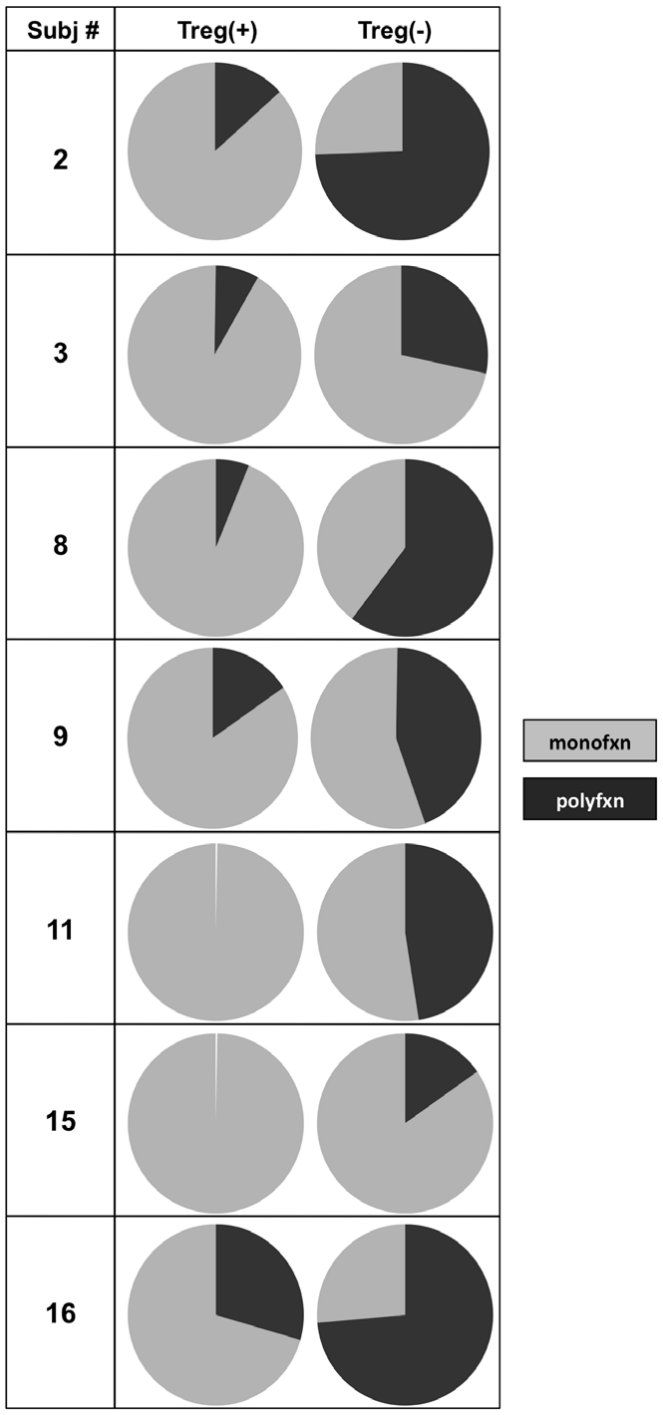
Proportion of monofunctional (monofxn) and polyfunctional (polyfxn) CD8^+^ T cells in 7 HIV-1 subjects. The polyfunctional vaccine response to Gag peptide in each of the 7 HIV-1-infected subjects is shown before [Treg(+)] and after [Treg(−)] Treg depletion.

We next evaluated polyfunctionality of the vaccine response itself in both the Treg(+) and Treg(−) samples by looking at the total vaccine response. As described earlier, the total vaccine response is the difference of the post-vaccine from the pre-vaccine response, and represents the vaccine-induced change in CD8^+^ T cell immune mediator secretion from baseline. Figure 6 shows the difference in polyfunctionality of the total vaccine response in the Treg(+) and Treg(−) samples. Upon Treg depletion, the CD8^+^ T cells were more likely to secrete 3–4 immune mediators, and the difference in the pattern was significant (P = 0.029) (Fig. 6). The average post-vaccine percentage of CD8^+^ T cells secreting at least two immune mediators more than doubled after removal of Treg. The samples containing Treg had a greater percentage of monofunctional CD8^+^ T cells post-vaccine. The CD8^+^ T cells expressed more CD107a and IFNγ (in 10/31 combinations of immune mediators) in response to Gag peptide as compared to the other mediators. Figure 7 shows that in all of 7 subjects in whom functional assays were done, there was an increase in the proportion of polyfunctional CD8^+^ T cells after Treg depletion.

Since we used two methods for Treg depletion, we asked whether polyfunctional responses of CD8^+^ T cells were influenced by the method used. Samples obtained from either method showed a trend towards more polyfunctionality with the removal of Treg despite the difference in the technique (P = 0.100 when using magnetic beads and P = 0.143 when using a cell sorter, data not shown).

**Figure 8:**
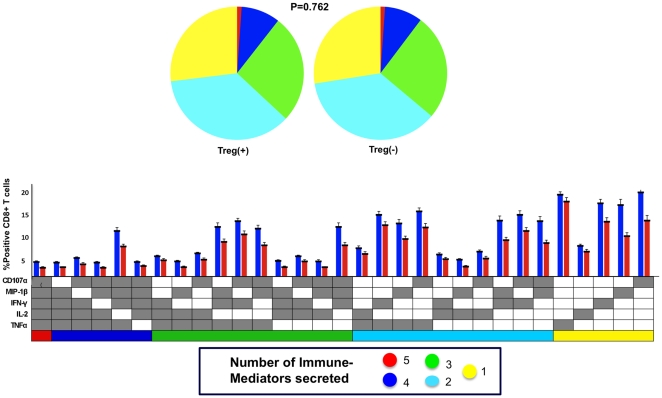
Comparison of response to *Staphylococcus* enterotoxin B (SEB) in samples with (Treg+) and without Treg (Treg−). Permutation comparison test showed no difference in the proportion of polyfunctional CD8^+^ T cells after Treg depletion. (P = 0.762; N = 7)

We then determined whether the Treg-mediated inhibition of polyfunctional responses was specific to the Gag peptide by examining CD8^+^ T cell immune responses to the positive control, SEB, since such superantigens have also been shown to induce regulatory T cells in vivo. [21] Not only did the CD8^+^ T cells secrete higher levels of the immune mediators, they also showed significant polyfunctional response to SEB. (Fig. 8) However, Treg depletion did not result in any significant change in the polyfunctionality of the cells (P = 0.762), implying specificity of the Treg suppressive function to the Gag peptide.

## Discussion

The goal of therapeutic vaccinations in HIV-1 infection is to provide enough antigenic stimulation so that the subject can mount an immune response in order to effectively control viral replication without the presence of an antiretroviral regimen. Recent studies of HIV-1-infected individuals with natural immune control have shown that an effective immune response is characterized by the presence of a polyfunctional CD8^+^ T cell response to the virus. [13], [14], [16], [20] Since the control of HIV-1 has been shown to be more dependent on the quality and specificity of the CD8^+^ T cell response rather than the quantity of the response alone, [15] we evaluated CD8^+^ T cell polyfunctional response in a previously completed DC-HIV-1 peptide vaccine trial. [12] Interestingly, the increased monofunctional response after vaccination correlated with the modest IFNγ response detected using the ELISPOT assay. However, although assessing single cell IFNγ production showed that there were transient and modest responses to the vaccine peptides, permutation comparison analysis showed no significant difference in the CD8^+^ T cell polyfunctional response post-vaccine. In fact, it appeared that the CD8^+^ T cell response to Gag peptide was more polyfunctional before the vaccine was given.

The minimal immunologic response to this DC-peptide vaccine could be due to its low immunogenicity. We examined an alternative hypothesis that Treg activity affected immunologic response in the trial. Depletion of Treg in the cord blood of HIV-1-exposed-uninfected neonates resulted in the augmentation of CD4^+^ and CD8^+^ T cell HIV-1-specific immune responses. [22] Also, Treg isolated from HIV-1-infected patients have been shown to suppress cytokine secretion. [2] Because of the major role that Treg, especially the antigen-specific Treg induced in the periphery, play in the immune response to chronic infections, it was important for us to delineate the effects of Treg in this particular study. The objective was to assess whether Treg activity should be a co-factor in the evaluation of immune-based therapeutic trials in HIV-1.

In our study, there was a trend of increased Treg frequency observed in a majority of subjects after DC-HIV peptide vaccination, with a modest median increase of 30% in the 12 subjects. Sixty four percent of vaccine non-responders by ELISPOT assay had increases in Treg frequency compared to 83% of ELISPOT assay responders. As Treg can accumulate in the lymphoid tissues, [2], [10] the modest increase in Treg frequency in peripheral blood as well as the lack of correlation between Treg frequency and vaccine response detected by ELISPOT assay could be due to migration of the expanded Treg population to the lymph nodes where T cells are being activated by the vaccine. Interestingly, the CD8^+^ T cell polyfunctional response appeared greater prior to the vaccine, suggesting that the increase in Treg frequency and Treg activity, could have affected the ability of CD8^+^ T cells to secrete cytokines. It is also important to note that when we checked the Treg frequencies 3 months after the vaccine was given, the levels decreased to baseline or lower than baseline in 14/14 subjects evaluated (data not shown) suggesting that the increased frequencies were vaccine related.

More important than the Treg frequency is the suppressive function, which we have shown to have an effect on the vaccine response, as evinced by the increased polyfunctionality of the CD8^+^ T cell response to Gag peptide after Treg depletion. Since the subjects have all been virally suppressed on a stable ART regimen, there is not enough antigenic stimulation, which is believed to be necessary for continuous anti-HIV-1 effector function. [23], [24] Upon receipt of the DC vaccine, it is likely that the Treg population was initially activated and expanded, as has been shown in mouse models. [25] Indeed, in the study by O'Gorman et al. [25], antigen-specific memory CD4^+^ T cells only acquire responsiveness after repeated antigenic stimulation. In our study, the high expression levels of the IL-2 receptor α chain, CD25, on Treg could have allowed them to sequester increased levels of IL-2 produced by the T cells upon antigen recognition. Thus, although our DC vaccine was able to elicit an immune response after the second dose, the increased expansion of Treg after the first dose might have significantly limited the total CD8^+^ T cell immune activity, leading to the modest and transient response.

The specific mechanism by which the Treg exerted suppression of polyfunctional responses of CD8^+^ T cells remains to be determined. However, it should be noted that there was no change in the expression of CTLA4 and GITR expression in Treg post-vaccine, suggesting that there could have been other mechanisms of suppression that were increased after vaccine administration leading to the increased suppressive function. Antigen-specific induced Treg such as type 1 regulatory T cells (Tr1) mediate suppression by IL10 secretion, which we were not able to test. Indeed, Lopez et al., [26] point to evidence indicating that the pre-treatment with immunoinhibitory drugs improves immune responses of cancer patients to DC therapy mainly because these drugs eliminate the Treg population. As the removal of the entire Treg population will also lead to autoimmunity [1], it is important to initially determine which specific Treg mechanism is responsible for immune suppression, and then, define the Treg subsets mediating suppression. In the context of enhancing CD8^+^ T cell responses against HIV-1, the goal would be inhibiting Treg suppression without causing a significant manifestation of autoimmunity.

Since polyfunctional T cell responses have been associated with better immunologic control of HIV-1, as shown in elite controllers, [15], [16] Treg appear to play a significant role in inhibiting effective immune responses to the virus in untreated patients. However, this inhibition may be beneficial for the host since persistent immune response will lead to persistent immune activation, which has been shown as a predictor of HIV-1 disease progression. [8] Moreover, activated T cells will also be easily targeted by the virus, thus contributing to progressive infection. Indeed, lower numbers of Treg have been associated with higher levels of immune activation in HIV-1 infection. [6], [27] Treg therefore seem to be maintaining a balance between persistent infection and decreased immune activation. As the CD4^+^ T cell population becomes depleted later in the disease, untreated patients would experience both increased immunosuppression and immune activation due to the eventual Treg depletion. Although Treg may not be exerting a considerable effect in virally suppressed patients on ART, an increase in the immune response to the virus, such as after administration of a therapeutic vaccine, may tilt this delicate balance held in check by Treg towards a proinflammatory T cell response. This will lead the Treg to counteract by increasing their frequency and suppressive effects thus preventing any resulting immune activation.

We believe that this is the first study to assess Treg activity in HIV-1 therapeutic vaccines. Although we were able to evaluate Treg frequencies in the 17 subjects who received the vaccine, we were only able to evaluate polyfunctional response in 7 subjects. It is important to note, however, that in all the 7 subjects, increased polyfunctionality was observed after Treg depletion implying that the DC vaccine did indeed increase polyfunctional CD8^+^ T cell activity but was masked by Treg expansion and increased suppressive function. Further research should be conducted to investigate the role of Treg in other immunotherapeutic trials for HIV-1. The information from these studies can guide investigators in developing better strategies to enhance HIV-1 immune control.
